# Photoluminescent Nanomaterials for Medical Biotechnology

**DOI:** 10.32607/actanaturae.11180

**Published:** 2021

**Authors:** E. L. Guryev, S. Shanwar, A.V. Zvyagin, S. M. Deyev, I. V. Balalaeva

**Affiliations:** Lobachevsky State University of Nizhny Novgorod, Nizhny Novgorod, 603022 Russia; Shemyakin-Ovchinnikov Institute of Bioorganic Chemistry, Russian Academy of Sciences, Moscow, 117997 Russia; I. M. Sechenov First Moscow State Medical University, Moscow, 119991 Russia

**Keywords:** photoluminescent nanomaterials, biotechnological application, optical diagnostics and therapy, chemical sensors, quantum dots, gold clusters, carbon dots, nanodiamonds, porous silicon, up-conversion nanoparticles

## Abstract

Creation of various photoluminescent nanomaterials has significantly expanded the arsenal of approaches used in modern biomedicine. Their unique photophysical properties can significantly improve the sensitivity and specificity of diagnostic methods, increase therapy effectiveness, and make a theranostic approach to treatment possible through the application of nanoparticle conjugates with functional macromolecules. The most widely used nanomaterials to date are semiconductor quantum dots; gold nanoclusters; carbon dots; nanodiamonds; semiconductor porous silicon; and up-conversion nanoparticles. This paper considers the promising groups of photoluminescent nanomaterials that can be used in medical biotechnology: in particular, for devising agents for optical diagnostic methods, sensorics, and various types of therapy.

## INTRODUCTION

In recent decades, there has been a qualitative shift in medicine towards more precise and personalized treatment through a combination of early diagnosis, therapy, and subsequent monitoring of the course of a disease. This approach is called theranostics. Nanotechnology – in combination with optical, acoustical and other methods of non-invasive application – occupies a dominant niche in this area. Nanoparticles are able to successfully combine several functions thanks to their unique properties, such as the programmability of physical and chemical characteristics, presence of reactive functional groups, a large surface to volume ratio, and optimal size. These features allow nanoparticles to act not only as independent therapeutic and/or contrast agents and delivery vehicles, but also as a platform for creating multifunctional complexes. In this context, optically active nanoparticles open up wide possibilities for the visualization of target cells or subcellular structures, in combination with a simultaneous targeted therapeutic effect.

One of the groups of nanomaterials used in optical diagnostics methods, sensorics, and therapy is the Photoluminescent Nanomaterials for Medical Biotechnology E. L. Guryev1, S. Shanwar1, A.V. Zvyagin1,2,3, S. M. Deyev2,3, I. V. Balalaeva1* 1Lobachevsky State University of Nizhny Novgorod, Nizhny Novgorod, 603022 Russia 2Shemyakin-Ovchinnikov Institute of Bioorganic Chemistry, Russian Academy of Sciences, Moscow, 117997 Russia 3I. M. Sechenov First Moscow State Medical University, Moscow, 119991 Russia *E-mail: irin-b@mail.ru Received August 30, 2020; in final form, October 12, 2020 DOI: 10.32607/actanaturae.11180 Copyright c 2021 National Research University Higher School of Economics. This is an open access article distributed under the Creative Commons Attribution License,which permits unrestricted use, distribution, and reproduction in any medium, provided the original work is properly cited. plasmon-resonance particles of gold, silver, and other metals. They have been proposed as a basis for a number of sensors for the qualitative and quantitative determination of various chemical compounds and biological macromolecules, as well as agents for visualizing and affecting target cells [1, 2, 3]. However, the overwhelming majority of the solutions rely upon the use of photoluminescent nanomaterials (PLNMs). Depending on their chemical structure, shape and size, the properties of such materials differ significantly, making them a suitable means to solving a wide range of practical problems. Nowadays, the most widely used in biomedical research are quantum dots, small gold clusters, carbon dots, nanodiamonds, semiconductor porous silicon, and up-conversion nanoparticles.

In this paper, we considered the PLNM groups that are of interest as a basis for devising agents for medical biotechnology: in particular, for optical diagnostic methods, sensorics, and various types of therapy.

## Quantum dots

**Fig. 1:**
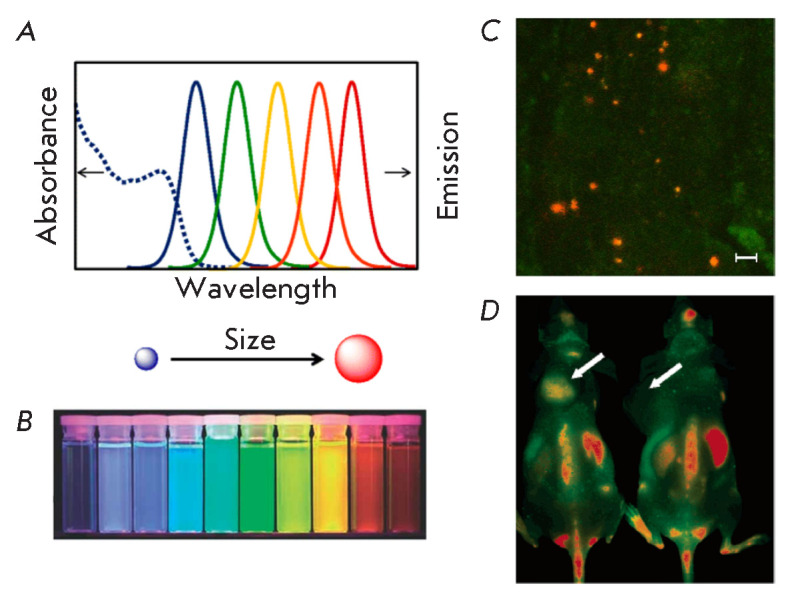
(*A*) – Size-dependence of the CdSe/ZnS QD fluorescence emission spectrum. Adapted from [9] with permission from the copyright holder: c 2017 by the authors. Licensee MDPI, Basel. (*B*) – Fluorescence photograph of QD suspensions irradiated with ultraviolet light (emission maxima at 443, 473, 481, 500, 518, 543, 565, 587, 610, and 655 nm). Adapted from [10] with permission from the copyright holder: John Wiley and Sons. c 2010 WILEY-VCH Verlag GmbH & Co. KGaA, Weinheim. (*C*) – Visualization of targeted QD conjugates (QD-4D5scFv) in a xenograft SK-BR-3 tumor. The image was obtained by confocal fluorescence microscopy. Scale bar 10 μm. Adapted from [11] with permission from the copyright holder. c 2019 by the authors. Licensee MDPI, Basel. (*D*) –Intravital visualization of the distribution of targeted QD conjugates (QD705-RGD) in the body of a mouse carrying the U87MG xenograft tumor (indicated by an arrow). Mouse tissue autofluorescence is shown in green; QD fluorescent signal –in red. Adapted from [12] with permission from the copyright holder. c 2006 American Chemical Society

Quantum dots (QDs) are the most thoroughly studied PLNMs [4, 5, 6]. They are inorganic nanocrystals usually consisting of elements of the II and VI or III and V groups and measuring in size between 2 and 10 nm. Most often, QDs are synthesized from such compounds as CdSe, CdS, CdTe, InAs, and GaAs with semiconductor properties in their bulk state. QDs possess photoluminescence (PL), with a quantum yield greater than 50% and a narrow symmetrical peak emission, whose position is determined by the particle size and composition (*Fig. 1*) [7, 8].

The PL properties of QDs are determined by the discrete energy levels that occur due to the restricted free motion of charge carriers (electrons and holes). Upon absorption of a quantum of exciting radiation, an electron enters a conduction zone with an excited state lasting from a few to tens of nanoseconds. A photon is emitted as a result of the radiative recombination of an electron-hole pair, and the photon energy corresponds to the difference between the highest hole and the lowest electron levels. Smaller particles have a larger energy difference between the corresponding levels, resulting in a higher energy of emitted photons and shorter wavelength.

In biomedicine, as a rule, QDs of improved structure are used, most often of improved core /shell structure, where the last forms from compounds with a similar crystal structure featuring the properties of wider-gap semiconductors [13 , 14, 15]. CdSe/ZnS QDs are used more often than others: they exhibit PL in the entire visible region of the spectrum depending on their particle size. The shell provides an increased PL quantum yield, contributes to QD surface stabilization, and prevents heavy metal ions from entering the environment, thereby reducing the toxic effect of such QDs relative to QDs without a shell [16, 17].

During synthesis, the surface of semiconductor QDs is covered with hydrophobic compounds, making them practically insoluble in water. To achieve colloidal stability and biocompatibility, the surface can be modified in various ways. One approach involves substituting hydrophobic surface ligands for hydrophilic ones or coating with amphiphilic compounds [18]. An alternative solution is to create an additional outer shell of either organic polymers [19, 20] or inorganic compounds (silicon oxide) [21]. The obtained QDs lack colloidal stability, which limits the scope of their poential use in biomedical applications. Various approaches to solving this problem have been described: however, a reliable and reproducible protocol has not yet been developed [22].

QDs have several useful photophysical properties, such as their high PL quantum yield and extinction coefficient, which enable one to visualize single nanoparticles; a wide absorption range and narrow symmetric PL emission peaks, making them useful in multiplex analysis [23]; long-term photostability, which allows for long-term tracking of individual molecules; and their wide multiphoton excitation range, which favourably distinguishes QDs from organic fluorophores [24]. In addition to the above-listed properties, many approaches to the surface functionalization of QDs and the attachment of various targeting/toxic modules specific to target molecules have been developed to date, enabling one to produce multifunctional complexes with the desired set of properties [25 , 26, 27].

Fluorescence imaging of cells, tissues, and organs is the main area of QD application (*Fig. 1*). With the more than 20 years it has been in use, QD imaging of cellular structures has become the standard approach. Specificity in staining certain cell components is achieved by using such targeting molecules as antibodies, peptides, nucleic acid fragments, and others. External modules are attached to particles by either chemical conjugation [28, 29], or by self-assembly, when illumina-specific adapters are added to streptavidin-biotin beads [30, 31], or by barnase-barstar [32, 33, 34, 35]. Such targeted complexes are actively used in optical microscopy, cell flow cytometry [36, 37], and immunohistochemical [38, 39] and enzyme immunoassay [40, 41].

A number of photophysical properties make QDs indispensable in the cases where organic fluorophores are of little use. In particular, QDs photostability enables one to study molecular dynamics: several studies have been performed to track receptors [42, 43, 44], integrins [45, 46], transport proteins [47], and membrane lipids [48].

One of the downsides of QDs is the intermittent nature of their PL (blinking) that occurs when one or both components of an exciton (electron and hole) hit the surface of the particle, which leads to the appearance of a charge on the particle and quenching of the PL as a result of nonradiative recombination [49]. In order to overcome this drawback, several methods have been designed that provide complete or partial blinking suppression [50, 51].

QDs are used to create sensors capable of assessing the quantitative content of various compounds in a medium. For this purpose, the changes in the emission characteristics (peak positions, intensity, polarization, kinetic parameters) associated with the attachment of target molecules to a QD surface are exploited [52, 53, 54, 56]. Many sensors using QDs as one of the participants in the Forster resonance energy transfer (FRET) pair have already been developed. Such systems are being successfully used to study the interaction between a ligand and a receptor, the specific detection of DNA sequences, and the detection of changes in protein molecule conformation [57, 58, 59].

Sensors are being actively developed that combine these approaches and are designed for a wide range of tasks such as detecting viruses and bacteria, determining the activity of enzymes and the presence of small organic molecules and various ions, and pH measuring [60, 61, 62].

The wide choice of synthesized QD components makes it possible to obtain particles with a PL emission in the near-IR region falling into the transparency window of biological tissue and to minimize light absorption and scattering [63]. The emission peaks of such particles remain narrow and symmetrical; and the QD size – within a few nanometers. Such characteristics enable one to actively use QD-based agents for noninvasive *in vivo *imaging of cells, tissues, and organs. Several studies have reported on the successful delivery of QD-based agents to tumor cells of various origins and to endotheliocytes of tumor vessels [12, 35, 64, 65, 66]. QDs emitting in the near-infrared region can effectively mark primary tumors and can be used to search for metastases [67, 68, 69], map lymph nodes [70, 71], study the vasculature [72], and track target tumor cells [73, 74].

QD complexes have an obvious therapeutic potential, particularly, in photodynamic therapy. When energy is transferred from QDs through organic dyes (FRET technology) or directly to an oxygen molecule, a pronounced photosensitizing effect can be observed [75, 76]. Finally, QDs can be used to monitor the efficiency of drug [77, 78] and nucleic acids delivery [79, 80]. Their use in clinical practice is constrained by their undesirable toxic effects associated with the presence of heavy metal ions and other hazardous substances (Cd, Pb, As, Te, Se) in their composition. The dynamics of their release into the surrounding environment mainly depends on their polymer coating. For instance, a biodegradable coating results in a significant release of components and obvious toxic effects [81, 82, 83]. A stronger polymer shell minimizes the side effects but greatly increases their retention time in the kidneys and spleen [84, 85, 86]. These features can significantly increase the risk of toxicity in clinical use. The way to overcome the described limitations, apparently, lies in the search for and design of PL agents that have a different chemical composition.

## Small gold clusters

**Fig. 2:**
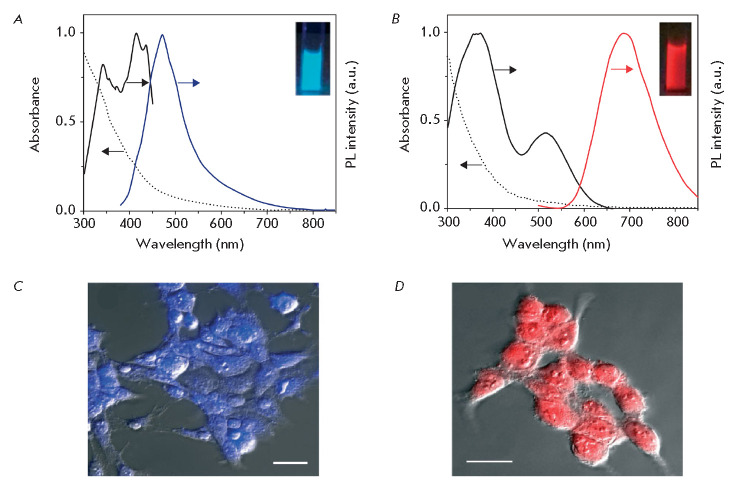
(*A*), (*B*) – Absorbance (dotted line), excitation (black), and PL emission (color) spectrum of gold nanoclusters. Insets – fluorescent photographs of gold nanocluster suspensions. (*C*), (*D*) – Visualization of HEK293 cells using gold nanoclusters emitting in the blue region of the spectrum (*C*) and gold nanoclusters coated with bovine serum albumin, emitting in the red region of the spectrum (*D*). Superposition of the transmitted light image and the PL signal of gold nanoclusters. Scale bar 50 μm. Adapted from [88] with permission from the copyright holder: John Wiley and Sons. c 2014 WILEY-VCH Verlag GmbH & Co. KGaA, Weinheim

Small gold clusters consisting of 2–100 atoms differ significantly in their properties from larger gold nanoparticles with a size of several nanometers or more. Gold clusters have intense fluorescence with a significant Stokes shift; a long-excited state lifetime; a high quantum yield; as well as photostability and biocompatibility. Their PL is determined by the transition of electrons between discrete molecular energy levels. The size and composition of the clusters determine the position of the PL emission peaks in a range from UV to IR (*Fig. 2A,BA,B*) [87, 88].

imaging agents, gold clusters allow one to achieve high image clarity and localization accuracy [96, 97]. Gold clusters coated with bovine serum albumin enable one to quickly and efficiently visualize tumor cells and whole tumors [98]. After entering the cells, small gold clusters are capable of emitting fluorescence for a long time (up to 28 days *in vitro*). Compared to QDs, they have lower cytotoxicity and insignificantly affect cell viability at comparable doses [99]. Their optical properties have made them an effective tool for such analytical methods as biomacromolecules detection [100] and tracking of drug distribution, as well as accumulation* in vivo *and *in vitro *[101].

Another interesting feature of small gold clusters is electroluminescence. So, they are widely used in the development of sensors [102]; in particular, for DNA and microRNA detection. One such development is a proposed biosensor for the detection of peroxidase genes using fluorescent gold clusters as a label [103].

Small gold clusters can also be used for a targeted delivery of the drugs attached to their surface. Effective delivery and controlled release of anticancer drugs (doxorubicin, cisplatin, captopril, and 6-mercaptopurine) using gold clusters encapsulated in dendrimers has been demonstrated in [104]. Gold clusters can also be used in gene therapy, providing systemic gene delivery and the visualizing of intracellular transport. As vectors, they favourably distinguish themselves by their low cytotoxicity, good photostability, and lack of an immune response [105].

Another interesting property of gold clusters is their radiosensitizing ability thanks to a high ionizing radiation absorption coefficient that is significantly higher than that of organic molecules [106, 107]. Being able to increase the radiosensitivity of tumor cells *in vivo* enables the clusters to increase the therapeutic efficacy of radiation therapy by locally increasing the Au concentration in the tumor [108].

The use of fluorescent gold clusters as contrast agents is “hindered” by a broad peak of PL emission, which makes it difficult to use several agents simultaneously [88]. Also, the problem related to the safety of nanomaterials made of gold and other noble metals remains unresolved. There is evidence that small gold clusters cause oxidative stress; disruption of the mitochondrial function; have a negative effect on nucleic acids, as well as on the level of proinflammatory cytokines; induce liver destruction, etc. [3, 109, 110]. On the other hand, the variety of structures and compositions of the agents based on small gold clusters used in these studies prevent us from drawing any definitive conclusion regarding the specific reasons behind these negative consequences.

## Carbon dots

Carbon dots (C-dots) are clusters of carbon atoms 2–8 nm in size with photoluminescent properties. They contain a significant amount of hydrogen and oxygen atoms, as well as traceable amounts of nitrogen, and can be of either amorphous (carbon in sp2- and sp3-hybridization) or graphene structure (sp2-hybridized atoms) [111, 112]. The advantages of C-dots are their photostability, wide surface modification capabilities, and low production cost, since they can be obtained using chemical treatment from the soot of many carbon- containing materials, including those of plant origin. [113, 114, 115].

**Fig. 3:**
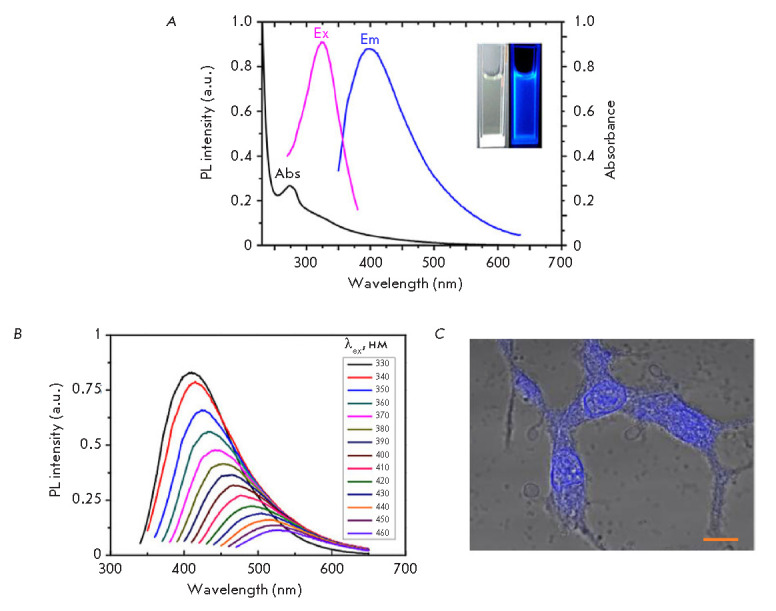
(*A*) – Spectrum of absorbance, excitation and PL emission of C-dots. Inset – brightfield and fluorescent photographs of suspensions of carbon dots. (*B*) – PL emission spectrum of C-dots upon excitation by light with different wavelengths. Adapted from [118] with permission from the copyright holder: c 2019 The Authors, Royal Society Publishing. (*C*) – Visualization of 293T cells using nitrogen-doped C-dots. Superimposed image in transmitted light and PL signal of carbon dots. Scale bar 10 μm. Adapted from [119] with permission from the copyright holder: Dove Medical Press Ltd. c 2016 Informa PLC, London

C-dots are characterized by a bright PL in a range of 300–500 nm determined by the defects in the particle surface, exciton recombination, and quantum-size effects (*Fig. 3A,B*). The absence of toxicity allows us to count on the widespread use of carbon dots in biomedicine, as has been indicated in many studies [114, 116, 117].

Carbon dots are effectively used as fluorophores in the development of sensors, in particular, to determine the metal ion content. Adding selective ligands makes it possible to create sensors for the Ag+, Al3+, Zn^2+^, Hg^2+^, and Cu^2+^ ions [120, 121, 122, 123, 124]. Connecting carbon dots (PL in the blue region) with quantum dots (PL in the red region) and coating with bovine serum albumin has given us a ratiometric sensor for the supersensitive determination of copper ions [125]. C-dots are successfully used to create highly sensitive systems for the immunofluorescence and enzyme immunoassay of various antigens [126, 127]. Thanks to the FRET technology, a pH-sensitive probe based on C-dots and a pH-sensitive dye (FITC) acting as an acceptor has been developed [128, 129]. The possibility to use C-dots in ratiometric complexes for assessing intracellular temperature has been demonstrated. Complexes of two types of carbon clusters differing in their PL emission spectrum that are thermosensitive in a range from 15 to 90°C and stable at pH values ranging from 4 to 9 and can be used for cell temperature mapping [130].

Apart from sensoring, C-dots are also used as drug carriers. Particularly, conjugates with the antitumor drug oxaliplatin have been obtained by covalent attachment to their modified surface [131]. An alternative drug delivery system is conjugates of C-dots and gold nanorods having pH-sensitive bonds. Such conjugates demonstrate an active release of bound doxorubicin upon changes in pH and exposure to radiation. The functionalization of such conjugates with folic acid has made it possible to create a theranostic complex suitable both for efficient visualization of tumor cells and targeted drug delivery with controlled release [132]. The targeting action of folic acid makes it possible to detect even single tumor cells [133].

Another theranostic application of C-dots has been complexes including organosilica nanospheres. These spheres are mesoporous, and as so they can include anticancer drugs in their composition, making the complexes capable of pH-dependent drug release (doxorubicin) and photothermal activity upon irradiation in the near-IR range [134].

Being non-toxic and biocompatible, C-dots open up prospects for use as an alternative to semiconductor quantum dots: however, their photophysical properties need to be modified to shift the PL emission maxima to the near-IR range [135, 136].

## Nanodiamonds

**Fig. 4:**
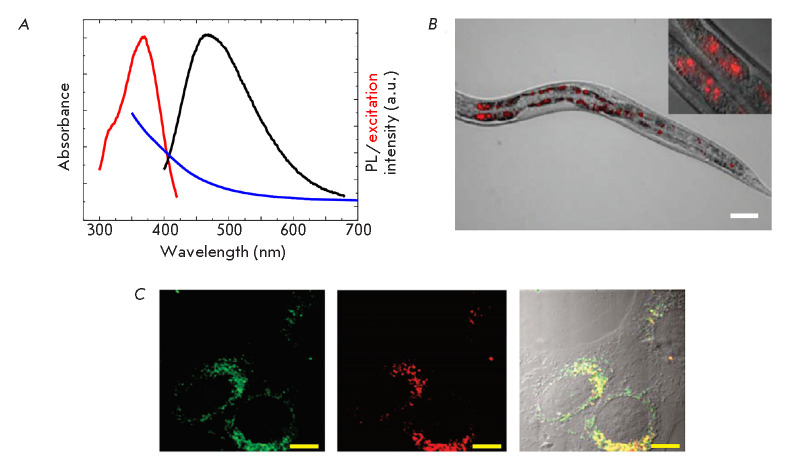
(*A*) – Absorbance (blue), excitation (emission at 490 nm), and PL emission spectrum of nanodiamonds. Adapted from [145] with permission from the copyright holder: IOP Publishing. c Copyright 2020 IOP Publishing, Bristol. (*B*) – Visualization of the intestines of the free-living worms *Caenorhabditis elegans *using nanodiamonds coated with bovine serum albumin. Inset – an enlarged view of intestinal cells containing nanodiamonds. The overlay images were obtained by the method of differential interference contrast and epiluminescent images in a range above 600 nm, with excitation in a range of 510–560 nm. Scale bar 50 μm. Adapted from [146] with permission from the copyright holder: American Chemical Society. c Copyright (2010) American Chemical Society. (*C*) –Visualization of HeLa cells using nanodiamonds. From left to right: confocal fluorescence image of LysoTracker-stained liposomes obtained in the range of 500–30 nm; confocal fluorescent image of nanodiamonds obtained in the range of 600–50 nm; overlay images. Scale bar 10 μm. Adapted from [147] with permission from the copyright holder: American Chemical Society. c Copyright (2009) American Chemical Society

Another type of carbon nanomaterials, nanodiamonds (NDs), has similar photoluminescent properties [137, 138]. NDs are composed of carbon sp3 hybridization atoms assembled into a crystal lattice of cubic syngony. The defects in the lattice structure that form localized excited states upon absorption of light quanta in the visible range cause NDs to be photoluminescent [139] (*Fig. 4A*). For this purpose, nanodiamond particles are doped with nitrogen atoms that form local defects of various types during synthesis [140] and the position of the emission maxima and their intensity are determined by the types of defects and the total amount of nitrogen doped. In particular, negatively charged nitrogen vacancies (NV–) cause a PL that is located in the 650–700 nm region, which is most preferable for bioimaging [141, 142, 143, 144].

NDs are currently being considered as a promising system for targeted drug delivery characterized by high delivery efficiency and low toxicity [148, 149, 150]. There are many potential biological and medical ND applications, including use in biocompatible composites and implants, targeted drug delivery, biosensor components, and as stable solid carriers for peptide synthesis (*Fig. 4B,C*). ND-based imaging and therapy helps in early diagnosis, treatment, and effective prevention of several diseases. The imaging methods make it possible to effectively determine the stage of a disease, carry out non-invasive monitoring of the effectiveness of treatment, and, as emphasized, predict the duration and degree of remission [151].

Zurbuchen *et al*. have demonstrated the subcellular multimodal imaging technique (using optical and electron microscopy) to facilitate the localization of NDs having fluorescent NV-centers. Thanks to their PL properties, the possibility of their use as agents for diagnosing nervous system diseases has been shown [152].

Having a large surface area, NDs are well suited for drug loading and functionalization. For instance, Huang* et al*. have demonstrated effective attachment of doxorubicin to nanodiamonds, with its subsequent release. It has been found that this compound is less toxic to normal cells and exhibits a higher activity against human colorectal cancer cells than free doxorubicin. The prolonged release ensures the required drug concentration at a lower administered dose [153]. It has been shown that clusters of nanodiamonds are able to enclose the drugs being delivered to isolate the delivered agent from healthy cells, allowing for most of the administered dose to reach the target area, increasing the healing effect [154].

Nanodiamonds are also considered as a promising tool for gene delivery in order to significantly increase gene therapy effectiveness. For instance, efficient delivery and subsequent expression of the green fluorescent protein gene has been demonstrated with spiky NDs as a carrier [155]. Another interesting direction in this respect is regenerative tissue engineering. Yang* et al*. have developed a polymer-based nanocomposite frame containing NDs to support the growth and differentiation of osteoblasts, as well as their enhanced biomineralization to stimulate bone formation *in vitro* [156].

Despite the obvious advantages of nanodiamonds, their practical application is limited by the laboriousness associated to their synthesis. They also require a solution to the aggregation problem and correction of their PL properties.

## Semiconductor porous silicon nanoparticles

**Fig. 5:**
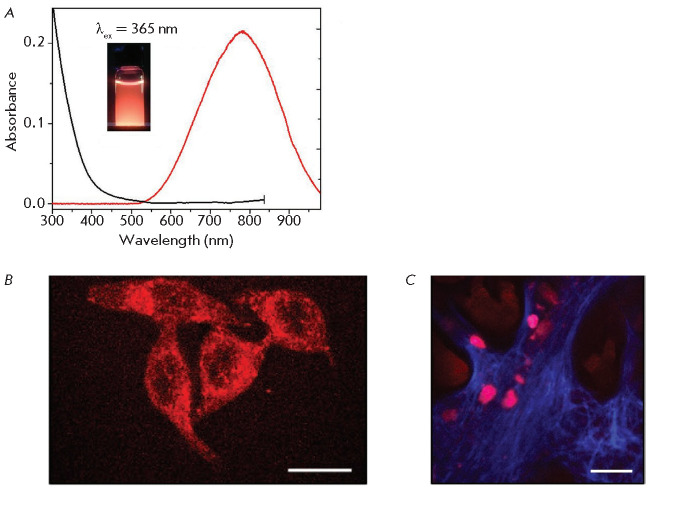
(*A*) – Spectrum of absorbance and PL emission of porous silicon nanoparticles upon excitation by 365-nm light. Inset – photograph of a colloidal solution of porous silicon nanoparticles irradiated with 365-nm light. (*B*) – HeLa cells labeled with targeted conjugates based on porous silicon nanoparticles. The image was obtained by two-photon microscopy at an exciting radiation power of 10 mW. Scale bar 15 μm. (*C*) –*In vivo *image of a xenograft HeLa tumor after injection of targeted conjugates based on porous silicon nanoparticles, obtained by two-photon microscopy. Scale bar 75 μm. Adapted from [162] with permission from the copyright holder: John Wiley and Sons. c 2017 WILEY-VCH Verlag GmbH & Co. KGaA, Weinheim

The fluorescent properties of semiconductor porous silicon nanoparticles (PSiNPs), like those of QDs, depend on quantum-size effects. These particles are biocompatible, biodegradable, and have low toxicity [157, 158]. The position of their PL emission maxima in the visible or near-IR region (*Fig. 5*) depends on the particle size and modification of their surface [138, 159, 160]. Large silicon particles that are not direct-gap semiconductors have a very low PL yield. On the contrary, particles up to 5 nm in diameter exhibit the properties of direct-gap semiconductors and bright PL, which, nonetheless, does not reach that of QDs [161].

The large content of silicon in the Earth’s crust significantly reduces the cost of synthesizing silicon nanoparticles, in comparison to other inorganic nanomaterials. PSiNPs have been used to create effective sensors to measure the pH level; the concentration of heavy metals, carbohydrates, pesticides, antibiotics, and other compounds [163, 164, 165]. Long-term monitoring of PSiNPs biodistribution in living organisms is possible thanks to their PL emission in the near-IR range [158]. The attachment of a protein or other targeting modules to PSiNPs makes it possible to obtain nanocomplexes both for specific visualization of cells and subcellular structures, as well as for whole-body imaging (*Fig. 5*) [162, 166, 167, 168].

PSiNPs have been successfully used for the delivery and controlled pH-dependent release of drugs; in particular, doxorubicin [169]. They are capable of inducing a photothermal effect; in particular, heating a tumor tissue to 60°C when irradiated with a 1064 -nm laser beam to induce apoptosis and angiogenesis suppression* in vivo *[170]. Their porous structure makes them easy for drug-loading, e.g., by the capillary method when it is enough to immerse a particle in a concentrated solution of a drug [171, 172]. The PSiNPs surface in most cases has a negative surface charge, enabling absorption of positively charged molecules, such as immunoglobulin-binding protein A [173]. It is the absorption principle that makes controlled delivery of small protein molecules possible. However, due to weak drug/particle interactions, PSiNPs provide for only rapid unloading, as opposed to long unloading periods when a drug is covalently bound to the carrier [174]. On the other hand, the hydroxylated pore surface makes it possible to covalently load drugs, particularly, doxorubicin, with its subsequent release [175]. Binding drugs to porous silicon particles improves their solubility [176, 177, 178], increases their biostability [179], as well as the ability of drugs to penetrate the body’s biological barriers.

PSiNPs, it is worth noting the problem of achieving bright PL in the transparency window of a biological tissue. It is possible to shift the PL emission maximum to the near-IR region by increasing the particle size, but this will lead to a simultaneous significant decrease in the PL yield. In addition, the problem of obtaining stable colloidal aqueous PSiNPs solutions resistant to oxygen has not been completely solved [138].

## Up-conversion nanoparticles

The significant autofluorescence of biological tissues complicates the registration of a target PL signal from different labels and probes [180, 181]. This is especially important in intravital imaging of individual cells or tissues of the body, where the level of autofluorescence is the main limitation to imaging sensitivity. The solution to this problem has been facilitated by studying up-conversion nanoparticles (UCNPs) that are inorganic nanocrystals consisting of an optically inert host matrix (NaYF_4_, Y_2_O_3_, NaPrF_4_, La_2_O_3_, Lu_2_O_3_, LuPO_4_, GdVO_4_, NaGdF_4_) and optically active lanthanide ions acting as luminescence centers [182, 183]. The best-studied among them are the NaYF_4_:Yb^3+^:Er^3+^/ Tm^3+^ UCNPs actively used in biomedical applications [137, 184, 185].

**Fig. 6:**
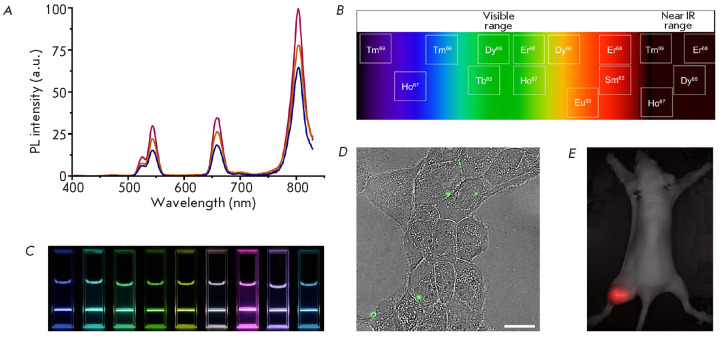
(*A*) – PL emission spectrum of NaYF4:Yb,Er,Tm UCNP upon excitation with 980-nm light of varying power. (*B*) – Dependence between dopant ion type and UCNP radiation wavelength. Adapted from [187] with permission from the copyright holder: Dove Medical Press Ltd. c 2019 Informa PLC, London. (*C*) – Photographs of various colloidal UCNP solutions irradiated with 980-nm light. Adapted from [188] with permission from the copyright holder: John Wiley and Sons. c 2013 WILEY-VCH Verlag GmbH & Co. KGaA, Weinheim. (*D*) – Visualization of SK-BR-3 cells with targeted NaYF4:Yb,Er UCNP complexes. The overlay of the translucent image and PL signal in the range 420–840 nm was obtained using a wide-field fluorescence microscopy system. Scale bar 20 μm. (*E*) –Visualization of a xenograft SK-BR-3 tumor with theranostic complexes based on UCNP with the composition of NaYF4:Yb,Tm. Superposition of a brightfield image and PL signal in the range 485–31 nm, obtained using a laboratory imaging system

The unique UCNP optical properties result from the up-conversion phenomenon, a nonlinear optical process where a nanoparticle sequentially absorbs two or more low-energy photons and emits a high-energy photon of a shorter wavelength. The energy of exciting IR light is absorbed by the ions of the sensitizer (Yb^3+^) and is transmitted non-radiatively to the surrounding ions-sensitizers Yb^3+^ and the ions-activators Er^3+^ and/or Tm^3+^. The excited states of lanthanide ions are long-lived, making it possible to absorb more than one quantum of light with a subsequent energy transfer to the same activator ion. The energy accumulated on these ions causes them to transition to high energy levels. The return to their initial state is accompanied either by non-radiative energy transfer or by photon emission, with the energies exceeding the energy of the exciting light. The Er^3+^ and Tm^3+^ ions have several energy levels to provide several narrow emission peaks in the visible and IR spectral regions (*Fig. 6*) [186].

Thanks to their photophysical properties, UCNPs hold several advantages over other fluorophores used in biomedicine. The pronounced emission maxima make it possible to record the PL signal, clearly distinguishing it from tissue autofluorescence and the scattered excitation radiation. PL excitation by near-infrared light falling into the biological tissue transparency window makes it possible to achieve a greater visualization depth. When using thulium-doped UCNPs, the PL emission maximum is also in the near-IR region. The long PL lifetime (up to milliseconds) makes it possible to implement delayed detection optical schemes, increasing the SNR [189]. Finally, UCNPs have high chemical photostability and low toxicity [190, 191].

UCNP limitations include a lower radiation conversion coefficient (within 1–2%) if compared to linear fluorescent materials. As in the case of other nanomaterials, reliable and stable procedures for UCNP preparation, modification, and functionalization are required, as well as a study of the possible negative consequences of their application [183, 185, 192, 193]. Despite this, a lot of evidence has been accumulated showing that UCNPs can be successfully applied in the development of agents for optical and multimodal imaging [194, 195], sensors [196, 197], as well as for photodynamic and photothermal therapy [198, 199].

Nowadays, UCNPs are proving themselves to be not only excellent imaging agents for fluorescence diagnostics, but also a highly efficient platform for assembling multifunctional theranostic complexes [200, 201, 202]. Modifying their surface with immunoglobulin- and non-immunoglobulin targeting modules enables one to use UCNPs in high-precision optical diagnostics of oncological diseases. The possibility of using UCNPs for specific visualization of tumor cells and experimental tumors has been demonstrated in [190, 203, 204, 205, 206]. Attachment of the bifunctional targeted toxins specific to the tumor cells of a certain molecular profile to biocompatible UCNPs makes it possible to open the therapeutic potential of the designed complexes [207, 208] and use the advantages of combined therapy. It has been shown that the efficacy of therapeutic modules (β-emitter and targeted toxin) increases by more than two orders of magnitude when they are used as parts of a theranostic nanocomplex to attack tumor cells [209].

UCNPs allow one to use deeply penetrating IR radiation to excite PL with its subsequent transfer to an organic molecule-effector (in the case of photodynamic therapy) or to gold/silver nanoparticles (in the case of photothermal therapy). Several studies have demonstrated the significant photodynamic effect UCNP complexes combining small molecules (rose Bengal, riboflavin) [210] and phototoxic proteins (KillerRed, mCherry) [106, 211] have on tumor cells.

## CONCLUSION

The development of various nanomaterials with photoluminescent properties has significantly expanded the arsenal of approaches used in modern biomedicine. The unique photophysical properties of these new materials make it possible to significantly improve the sensitivity and specificity of diagnostic methods and also enable one to apply the theranostic approach to treatment using PL conjugates of nanoparticles and functional macromolecules. The size and surface properties of PL nanoparticles ensure efficient delivery of low-molecular-weight therapeutic agents of various natures, as well as biologically active macromolecules. Despite the positive features inherent in each type of the PL nanomaterials described above, it must be admitted that they also have a common downside that prevents their active introduction into widespread clinical practice and concerns the response of the immune system to the nanomaterials injected into the bloodstream for systemic delivery. The immune system cells that protect the body against foreign agents attack nanomaterials, and the latter fail to reach their target pathogenic cells and instead are quickly inactivated and accumulate in healthy tissues, primarily in the liver. This short circulation challenge has traditionally been solved by coating the nanomaterials with inert polymers to mask them from the immune system. These so-called stealth nanoagents, primarily liposomes, have been used recurrently over the past decades but have not become a cardinal solution to the problem. Recently, a fundamentally new approach has been proposed that makes it possible to significantly extend the circulation time of nanoagents and, as a consequence, to increase their therapeutic effect. The approach, called “cytoblockade of the mononuclear phagocytic system,” does not require any modification of the nanoparticles and consists in introducing a relatively small amount of antibodies against the body’s own red blood cells. As a result, the immune system “focuses” on attacking its own erythrocytes and for some time “ceases to see” the injected nanomaterials. During this time, the materials manage to locate target pathogenic objects and provide a therapeutic effect. An important characteristic feature of this approach is its versatility: i.e., independence of the nature, size, and other properties of the nanoparticles [212]. Ideologically close to this approach is the method in which “inert” nanoagents are first introduced into the body, triggering an attack by the immune system, and only after that are drug-loaded nanoparticles introduced [213]. Thus, it can be concluded that studies of the practical application of theranostic PL drugs should focus on a combination of highly effective, targeted nanoagents capable of detecting a pathogenic centre with high accuracy [214] and technologies that ensure their sufficiently long circulation time in the bloodstream.

## References

[1] Dykman L.A., Khlebtsov N.G. (2016). Biomaterials..

[2] Singh P., Pandit S., Mokkapati V.R.S.S., Garg A., Ravikumar V., Mijakovic I. (2018). Int. J. Mol. Sci..

[3] Azharuddin M., Zhu G.H., Das D., Ozgur E., Uzun L., Turner A.P.F., Patra H.K. (2019). Chem. Commun. (Camb.)..

[4] Chan W.C., Maxwell D.J., Gao X., Bailey R.E., Han M., Nie S. (2002). Curr. Opin. Biotechnol..

[5] Watson A., Wu X., Bruchez M. (2003). Biotechniques..

[6] Pleskova S., Mikheeva E., Gornostaeva E. (2018). Adv. Exp. Med. Biol..

[7] Ozkan M. (2004). Drug Discov. Today..

[8] Gao X., Yang L., Petros J.A., Marshall F.F., Simons J.W., Nie S. (2005). Curr. Opin. Biotechnol..

[9] Wen L., Qiu L., Wu Y., Hu X., Zhang X. (2017). Sensors..

[10] Mansur H.S. (2010). WIREs Nanomed. Nanobiotechnol..

[11] Kutova O.M., Guryev E.L., Sokolova E.A., Alzeibak R., Balalaeva I.V. (2019). Cancers..

[12] Cai W., Shin D.W., Chen K., Gheysens O., Cao Q., Wang S.X., Gambhir S.S., Chen X. (2006). Nano Lett..

[13] Hines M.A., Guyot-Sionnest P. (1996). J. Phys. Chem..

[14] Parra G.G., Ferreira L.P., Gonçalves P.J., Sizova S.V., Oleinikov V.A., Morozov V.N., Kuzmin V.A., Borissevitch I.E. (2018). Nanoscale Res. Lett..

[15] Wegner K.D., Dussert F., Truffier-Boutry D., Benayad A., Beal D., Mattera L., Ling W.L., Carrière M., Reiss P. (2019). Front. Chem..

[16] Baláž P., Baláž M., Dutková E., Zorkovská A., Kováč J., Hronec P., Kováč J.Jr., Čaplovičová M., Mojžiš J., Mojžišová G. (2016). Mater. Sci. Eng. C Mater. Biol. Appl..

[17] Modlitbová P., Pořízka P., Novotný K., Drbohlavová J., Chamradová I., Farka Z., Zlámalová-Gargošová H., Romih T., Kaiser J. (2018). Ecotoxicol. Environ. Saf..

[18] Wageh S., Maize M., Donia A.M., Al-Ghamdi A.A., Umar A. (2015). J. Nanosci. Nanotechnol..

[19] Pellegrino T., Manna L., Kudera S., Liedl T., Koktysh D., Rogach A.L., Keller S., Radler J., Natile G., Parak W.J. (2004). Nano Lett..

[20] Tomczak N., Liu R., Vancso J.G. (2013). Nanoscale..

[21] Goftman V.V., Aubert T., Ginste D.V., Van Deun R., Beloglazova N.V., Hens Z., De Saeger S., Goryacheva I.Y. (2016). Biosens. Bioelectron..

[22] Foubert A., Beloglazova N.V., Rajkovic A., Sas B., Madder A., Goryacheva I.Y., De Saeger S. (2016). Trends Anal. Chem..

[23] Rousserie G., Sukhanova A., Even-Desrumeaux K., Fleury F., Chames P., Baty D., Oleinikov V., Pluot M., Cohen J.H., Nabiev I. (2010). Crit. Rev. Oncol. Hematol..

[24] Durr N.J., Larson T., Smith D.K., Korgel B.A., Sokolov K., Ben-Yakar A. (2007). Nano Lett..

[25] Medintz I.L., Uyeda H.T., Goldman E.R., Mattoussi H. (2005). Nat. Mater.,.

[26] Smith A., Duan H., Mohs A., Nie S. (2008). Adv. Drug Del. Rev..

[27] Sperling R.A., Parak W.J. (2010). Philos. Trans. R. Soc. A. Math. Phys. Eng. Sci..

[28] Sukhanova A., Venteo L., Devy J., Artemyev M., Oleinikov V., Pluot M., Nabiev I. (2002). Lab. Invest..

[29] Jiang W., Mardyani S., Fischer H., Chan W.C.W. (2006). Chem. Mater..

[30] Yu Y., Duan S., He J., Liang W., Su J., Zhu J., Hu N., Zhao Y., Lu X. (2016). Oncol. Rep..

[31] Tomlinson I.D., Kovtun O., Crescentini T.M., Rosenthal S.J. (2019). Bioorg. Med. Chem. Lett..

[32] Nikitin M.P., Zdobnova T.A., Lukash S.V., Stremovskiy O.A., Deyev S.M. (2010). Proc. Natl. Acad. Sci. USA..

[33] Zdobnova T.A., Dorofeev S.G., Tananaev P.N., Vasiliev R.B., Balandin T.G., Edelweiss E.F., Stremovskiy O.A., Balalaeva I.V., Turchin I.V., Lebedenko E.N. (2009). J. Biomed. Opt..

[34] Zdobnova T.A., Stremovskiy O.A., Lebedenko E.N., Deyev S.M. (2012). PLoS One..

[35] Balalaeva I.V., Zdobnova T.A., Krutova I.V., Brilkina A.A., Lebedenko E.N., Deyev S.M. (2012). J. Biophotonics..

[36] Buranda T., Wu Y., Sklar L.A. (2011). Methods Mol. Biol..

[37] Kovtun O., Ross E.J., Tomlinson I.D., Rosenthal S.J. (2012). Chem. Commun. (Camb.)..

[38] Sun J.Z., Chen C., Jiang G., Tian W.Q., Li Y., Sun S.R. (2014). Int. J. Nanomedicine..

[39] Tang T., Zhang D.L. (2017). Oncol. Lett..

[40] Beloglazova N.V., Sobolev A.M., Tessier M.D., Hens Z., Goryacheva I.Y., De Saeger S. (2017). Methods..

[41] Suzuki M., Udaka H., Fukuda T. (2017). J. Pharm. Biomed. Anal..

[42] Dahan M., Lévi S., Luccardini C., Rostaing P., Riveau B., Triller A. (2003). Science..

[43] Lidke D.S., Nagy P., Heintzmann R., Arndt-Jovin D.J., Post J.N., Grecco H.E., Jares-Erijman E.A., Jovin T.M. (2004). Nat. Biotech..

[44] Madhankumar A.B., Mrowczynski O.D., Patel S.R., Weston C.L., Zacharia B.E., Glantz M.J., Siedlecki C.A., Xu L.C., Connor J.R. (2017). Acta Biomater..

[45] Echarte M.M., Bruno L., Arndt-Jovin D.J., Jovin T.M., Pietrasanta L.I. (2007). FEBS Lett..

[46] Arora N., Syed A., Sander S., Smith E.A. (2014). Phys. Biol..

[47] Bailey D.M., Kovtun O., Rosenthal S.J. (2017). Methods Mol. Biol..

[48] Chang J.C., Rosenthal S.J. (2012). ACS Chem. Neurosci..

[49] Efros A.L., Nesbitt D.J. (2016). Nat. Nanotechnol..

[50] Omogo B., Gao F., Bajwa P., Kaneko M., Heyes C.D. (2016). ACS Nano..

[51] Thomas E.M., Ghimire S., Kohara R., Anil A.N., Yuyama K.I., Takano Y., Thomas K.G., Biju V. (2018). ACS Nano..

[52] Susha A.S., Javier A.M., Parak W.J., Rogach A.L. (2006). Colloids Surf. A..

[53] Shang L., Zhang L., Dong S. (2009). Analyst..

[54] Generalova A.N., Oleinikov V.A., Sukhanova A., Artemyev M.V., Zubov V.P., Nabiev I. (2013). Biosens. Bioelectron..

[55] Zhang H., Zhang L., Liang R.P., Huang J., Qiu J.D. (2013). Anal. Chem..

[56] Kavosia B., Navaee A., Salimi A. (2018). Luminescence..

[57] Patolsky F., Gill R., Weizmann Y., Mokari T., Banin U., Willner I. (2003). J. Am. Chem. Soc..

[58] Guo Y., Sakonsinsiri C., Nehlmeier I., Fascione M.A., Zhang H., Wang W., Pöhlmann S., Turnbull W.B., Zhou D. (2016). Angew. Chem. Int. Ed. Engl..

[59] Yang L.H., Ahn D.J., Koo E. (2016). Mater. Sci. Eng. C Mater. Biol. Appl..

[60] Algar R.W., Tavares A.J., Krull U.J. (2010). Anal. Chim. Acta..

[61] Hu J., Wang Z.Y., Li C.C., Zhang C.Y. (2017). Chem. Commun. (Camb.)..

[62] Lesiak A., Drzozga K., Cabaj J., Bański M., Malecha K., Podhorodecki A. (2019). Nanomaterials (Basel)..

[63] Cassette E., Helle M., Bezdetnaya L., Marchal F., Dubertret B., Pons T. (2013). Adv. Drug. Deliv. Rev..

[64] Balalaeva I.V., Zdobnova T.A., Sokolova E.A., Deyev S.M. (2015). Russ. J. Bioorganic Chem..

[65] Akerman M.E., Chan W.C.W., Laakkonen P., Bhatia S.N., Ruoslahti E. (2002). Proc. Natl. Acad. Sci. USA..

[66] Gao X., Cui Y., Levenson R.M., Chung L.W.K., Nie S. (2004). Nat. Biotech..

[67] Helle M., Cassette E., Bezdetnaya L., Pons T., Leroux A., Plénat F., Guillemin F., Dubertret B., Marchal F. (2012). PLoS One..

[68] Jeong S., Jung Y., Bok S., Ryu Y.M., Lee S., Kim Y.E., Song J., Kim M., Kim S.Y., Ahn G.O. (2018). Adv. Healthc. Mater..

[69] Mangeolle T., Yakavets I., Lequeux N., Pons T., Bezdetnaya L., Marchal F. (2019). Photodiagnosis Photodyn. Ther..

[70] Si C., Zhang Y., Lv X., Yang W., Ran Z., Sun P. (2014). J. Surg. Res..

[71] Bakalova R., Zhelev Z., Nikolova B., Murayama S., Lazarova D., Tsoneva I., Aoki I. (2015). Gen. Physiol. Biophys..

[72] Wang H., Yang H., Xu Z.P., Liu X., Roberts M.S., Liang X. (2018). Pharmaceutics..

[73] Han H.S., Niemeyer E., Huang Y., Kamoun W.S., Martin J.D., Bhaumik J., Chen Y., Roberge S., Cui J., Martin M.R. (2015). Proc. Natl. Acad. Sci. USA..

[74] Zhang Z., Yuan Y., Liu Z., Chen H., Chen D., Fang X., Zheng J., Qin W., Wu C. (2018). ACS Appl Mater. Interfaces..

[75] Ipe B.I., Lehnig M., Niemeyer C.M. (2005). Small..

[76] Shen Y., Sun Y., Yan R., Chen E., Wang H., Ye D., Xu J.J., Chen H.Y. (2017). Biomaterials..

[77] Savla R., Taratula O., Garbuzenko O., Minko T. (2011). J. Control Release..

[78] Yang X., Zhang W., Zhao Z., Li N., Mou Z., Sun D., Cai Y., Wang W., Lin Y. (2017). J. Inorg. Biochem..

[79] Zhu H., Zhang S., Ling Y., Meng G., Yang Y., Zhang W. (2015). J. Control Release..

[80] Lin G., Chen T., Zou J., Wang Y., Wang X., Li J., Huang Q., Fu Z., Zhao Y., Lin M.C. (2017). Front. Pharmacol..

[81] Fan J., Sun Y., Wang S., Li Y., Zeng X., Cao Z., Yang P., Song P., Wang Z., Xian Z. (2016). Biomaterials..

[82] Yong K.T., Law W.C., Hu R., Ye L., Liu L., Swihart M.T., Prasad P.N. (2013). Chem. Soc. Rev..

[83] Sharma V.K., McDonald T.J., Sohn M., Anquandah G.A.K., Pettine M., Zboril R. (2017). Chemosphere..

[84] Yang R.S., Chang L.W., Wu J.P., Tsai M.H., Wang H.J., Kuo Y.C., Yeh T.K., Yang C.S., Lin P. (2007). Environ. Health. Perspect..

[85] Fitzpatrick J.A., Andreko S.K., Ernst L.A., Waggoner A.S., Ballou B., Bruchez M.P. (2009). Nano Lett..

[86] Carvalho S.M.D., Mansur A.A.P., Mansur H.S., Guedes M.I.M.C., Lobato Z.I.P., Leite M.F. (2017). Mater. Sci. Eng. C Mater. Biol. Appl..

[87] Zheng J., Zhang C.W., Dickson R.M. (2004). Phys. Rev. Lett..

[88] Palmal S., Jana N.R. (2014). WIREs Nanomed. Nanobiotechnol..

[89] Wen F., Dong Y., Feng L., Wang S., Zhang S., Zhang X. (2011). Anal. Chem..

[90] Wang Y., Chen J.T., Yan X.P. (2013). Anal. Chem..

[91] Liu M., Tang F., Yang Z., Xu J., Yang X. (2019). J. Anal. Methods. Chem..

[92] El-Sayed I.H., Huang X., El-Sayed M.A. (2006). Cancer Lett..

[93] Chen D., Luo Z., Li N., Lee J.Y., Xie J., Lu J. (2013). Adv. Funct. Mater..

[94] Xia F., Hou W., Zhang C., Zhi X., Cheng J., de la Fuente J.M., Song J., Cui D. (2018). Acta Biomater..

[95] Proshkina G., Deyev S., Ryabova A., Tavanti F., Menziani M.C., Cohen R., Katrivas L., Kotlyar A. (2019). ACS Appl. Mater. Interfaces..

[96] Wang C., Wang Y., Xu L., Shi X., Li X., Xu X., Sun H., Yang B., Lin Q. (2013). Small..

[97] Deyev S., Proshkina G., Ryabova A., Tavanti F., Menziani M.C., Eidelshtein G., Avishai G., Kotlyar A. (2017). Bioconjug. Chem..

[98] Zhang W., Ye J., Zhang Y., Li Q., Dong X., Jianga H., Wang X. (2015). RSC Adv..

[99] Wang X., Cai X., Hu J., Shao N., Wang F., Zhang Q., Xiao J., Cheng Y. (2013). J. Am. Chem. Soc.,.

[100] Qin L., He X., Chen L., Zhang Y. (2015). ACS Appl. Mater. Interfaces..

[101] Chen Z., Qian S., Chen J., Cai J., Wu S., Cai Z. (2012). Talanta..

[102] Li W., Chen X. (2015). Nanomedicine (Lond)..

[103] Zhou Y., Tang L., Zeng G., Chen J., Wang J., Fan C., Yang G., Zhang Y., Xie X. (2015). Biosens. Bioelectron..

[104] Wang H.H., Lin C.A.J., Lee C.H., Lin Y., Tseng Y.M., Hsieh C.L., Chen C.H., Tsai C.H., Hsieh C.T., Shen J. (2011). ACS Nano..

[105] Tao Y., Li Z., Ju E., Ren J., Qu X. (2013). Nanoscale..

[106] Liang L., Lu Y., Zhang R., Care A., Ortega T.A., Deyev S.M., Qian Y., Zvyagin A.V. (2017). Acta Biomaterialia..

[107] Kefayat A., Ghahremani F., Motaghi H., Amouheidari A. (2019). Nanomedicine..

[108] Hainfeld J.F., Smilowitz H.M., O’Connor M.J., Dilmanian F.A., Slatkin D.N. (2013). Nanomed..

[109] Lopez-Chaves C., Soto-Alvaredo J., Montes-Bayon M., Bettmer J., Llopis J., Sanchez-Gonzalez C. (2018). Nanomedicine..

[110] Raftis J.B., Miller M.R. (2019). Nano Today..

[111] Xu X., Ray R., Gu Y., Ploehn H.J., Gearheart L., Raker K., Scrivens W.A. (2004). J. Am. Chem. Soc..

[112] Sun Y.P., Zhou B., Lin Y., Wang W., Fernando K.A., Pathak P., Meziani M.J., Harruff B.A., Wang X., Wang H. (2006). J. Am. Chem. Soc..

[113] Himaja A.L., Karthik P.S., Singh S.P. (2015). Chem. Rec..

[114] Mishra V., Patil A., Thakur S., Kesharwani P. (2018). Drug Discov. Today..

[115] Boakye-Yiadom K.O., Kesse S., Opoku-Damoah Y., Filli M.S., Aquib M., Joelle M.M.B., Farooq M.A., Mavlyanova R., Raza F., Bavi R. (2019). Int. J. Pharm..

[116] Du J., Xu N., Fan J., Sun W., Peng X. (2019). Small..

[117] Nekoueian K., Amiri M., Sillanpää M., Marken F., Boukherroub R., Szunerits S. (2019). Chem. Soc. Rev..

[118] Li J., Tang K., Yu J., Wang H., Tu M., Wang X. (2019). R. Soc. Open Sci..

[119] Lu S.S., Guo S.S., Xu P.X., Li X.R., Zhao Y.M., Gu W., Xue M. (2016). Int. J. Nanomedicine..

[120] Algarra M., Campos B.B., Radotić K., Mutavdžić D., Bandosz T., Jiménez-Jiménez J., Rodriguez-Castellón E., Esteves da Silva J.C.G. (2014). J. Mater. Chem. A..

[121] Kim Y., Jang G., Lee T.S. ACS Ap.

[122] Zhang Z., Shi Y., Pan Y., Cheng X., Zhang L., Chen J., Li M.J., Yi C. (2014). J. Mater. Chem. B..

[123] Yuan C., Liu B., Liu F., Han M.Y., Zhang Z. (2014). Anal. Chem..

[124] Zhu A., Qu Q., Shao X., Kong B., Tian Y. (2012). Angew. Chem. Int. Ed..

[125] Qu Q., Zhu A., Shao X., Shi G., Tian Y. (2012). Chem. Commun..

[126] Zhu L., Cui X., Wu J., Wang Z., Wang P., Hou Y., Yang M. (2014). Anal. Methods..

[127] Wu Y., Wei P., Pengpumkiat S., Schumacher E.A., Remcho V.T. (2015). Anal. Chem..

[128] Shi W., Li X., Ma H. (2012). Angew. Chem. Int. Ed..

[129] Du F., Ming Y., Zeng F., Yu C., Wu S. (2013). Nanotechnology..

[130] Wang C., Hu T., Thomas T., Song S., Wen Z., Wang C., Song Q., Yang M. (2018). Royal Soc. Chem..

[131] Zheng M., Liu S., Li J., Qu D., Zhao H., Guan X., Hu X., Xie Z., Jing X., Sun Z. (2014). Adv. Mater..

[132] Liu Q., Xu S., Niu C., Li M., He D., Lu Z., Ma L., Na N., Huang F., Jiang H. (2015). Biosens. Bioelectron..

[133] Mewada A., Pandey S., Thakur M., Jadhav D., Sharon M. (2014). J. Mater. Chem. B..

[134] Singh R.K., Patel K.D., Mahapatra C., Kang M.S., Kim H.-W. (2016). ACS Appl. Mater. Interfaces..

[135] Liu J.J., Li D.W., Zhang K., Yang M.X., Sun H.C., Yang B. (2018). Small..

[136] Li Y., Bai G., Zeng S., Hao J. (2019). ACS Appl. Mater. Interfaces..

[137] Sreenivasan V.K., Zvyagin A.V., Goldys E.M. (2013). J. Phys. Condens. Matter..

[138] Montalti M., Cantelli A., Battistelli G. (2015). Chem. Soc. Rev..

[139] Aharonovich I., Greentree A.D., Prawer S. (2011). Nat. Photonics..

[140] Xing Y., Dai L. (2009). Nanomed. (Lond)..

[141] Vaijayanthimala V., Cheng P.Y., Yeh S.H., Liu K.K., Hsiao C.H., Chao J.I., Chang H.C. (2012). Biomaterials..

[142] Wu T.J., Tzeng Y.K., Chang W.W., Cheng C.A., Kuo Y., Chien C.H., Chang H.C., Yu J. (2013). Nat. Nanotechnol..

[143] Gerstenhaber J.A., Barone F.C., Marcinkiewicz C., Li J., Shiloh A.O., Sternberg M., Lelkes P.I., Feuerstein G. (2017). Int. J. Nanomedicine..

[144] van der Laan K., Hasani M., Zheng T., Schirhagl R. (2018). Small..

[145] Kharin A., Rogov A., Geloen A., Lysenko V., Bonacina L. (2016). J. Phys.: Conf. Series..

[146] Mohan N., Chen C.S., Hsieh H.H., Wu Y.C., Chang H.C. (2010). Nano Lett..

[147] Faklaris O., Joshi V., Irinopoulou T., Tauc P., Sennour M., Girard H., Gesset C., Arnault J.C., Thorel A., Boudou J.P. (2009). ACS Nano..

[148] Schrand A.M., Huang H., Carlson C., Schlager J.J., Ōsawa E., Hussain S.M., Dai L.. (2007). J. Phys. Chem. B..

[149] Vaijayanthimala V., Tzeng Y.K., Chang H.C., Li C.L. (2009). Nanotechnology..

[150] Huang Y.A., Kao C.W., Liu K.K., Huang H.S., Chiang M.H., Soo C.R., Chang H.C., Chiu T.W., Chao J.I., Hwang E. (2014). Sci. Rep..

[151] Fu C.C., Lee H.Y., Chen K., Lim T.S., Wu H.Y., Lin P.K., Wei P.K., Tsao P.H., Chang H.C., Fann W. (2007). Proc. Natl. Acad. Sci. USA..

[152] Zurbuchen M.A., Lake M.P., Kohan S.A., Leung B., Bouchard L.-S. (2013). Sci. Rep..

[153] Huang H., Pierstorff E., Osawa E., Ho D. (2007). Nano Lett..

[154] Sachdeva M.S. (1998). Expert Opin. Investig. Drugs..

[155] Chu Z., Miu K., Lung P., Zhang S., Zhao S., Chang H.C., Lin G., Li Q. (2015). Sci. Rep..

[156] Yang L., Webster T.J. (2014). IEEE Pulse..

[157] Canham L.T. (1995). Adv Mater..

[158] Park J.H., Gu L., von Maltzahn G., Ruoslahti E., Bhatia S.N., Sailor M.J. (2009). Nat. Mater..

[159] Chinnathambi S., Chen S., Ganesan S., Hanagata N. (2014). Adv. Healthc. Mater..

[160] Dasog M., De los Reyes G.B., Titova L.V., Hegmann F.A., Veinot J.G. (2014). ACS Nano..

[161] Takagahara T., Takeda K. (1992). Phys. Rev. B Condens. Matter..

[162] Kim D., Kang J., Wang T., Ryu H.G., Zuidema J.M., Joo J., Kim M., Huh Y., Jung J., Ahn K.H. (2017). Adv. Mater..

[163] Chu B., Wang H., Song B., Peng F., Su Y., He Y. (2016). Anal. Chem..

[164] Ma S.D., Chen Y.L., Feng J., Liu J.J., Zuo X.W., Chen X.G. (2016). Anal. Chem..

[165] Wang H., He Y. (2017). Sensors (Basel)..

[166] Erogbogbo F., Tien C.A., Chang C.W., Yong K.T., Law W.C., Ding H., Roy I., Swihart M.T., Prasad P.N. (2011). Bioconjug. Chem..

[167] Tolstik E., Osminkina L.A., Matthäus C., Burkhardt M., Tsurikov K.E., Natashina U.A., Timoshenko V.Y., Heintzmann R., Popp J., Sivakov V. (2016). Nanomedicine..

[168] Cao Z., Peng F., Hu Z., Chu B., Zhong Y., Su Y., He S., He Y. (2017). Nanoscale..

[169] Wang Q., Bao Y., Ahire J., Chao Y. (2012). Adv. Healthc. Mater..

[170] Yu X., Yang K., Chen X., Li W. (2017). Biomaterials..

[171] Bimbo L.M., Mäkilä E., Laaksonen T., Laaksonen T., Laaksonen P., Strommer K., Kauppinen E.I., Salonen J., Linder M.B., Hirvonen J., Santos H.A. (2011). Biomaterials..

[172] Foraker A.B., Walczak R.J., Cohen M.H., Boiarski T.A., Grove C.F., Swaan P.W. (2003). Pharm. Res..

[173] Schwartz M.P., Yu C., Alvarez S.D., Migliori B., Godin D., Chao L., Sailor M.J. (2007). Phys. Status Solidi A..

[174] Pastor E., Matveeva E., Valle-Gallego A., Goycoolea F.M., Garcia-Fuentes M. (2011). Colloid Surf. B..

[175] Wu E.C., Park J.H., Park J., Segal E., Cunin F., Sailor M.J. (2008). ACS Nano..

[176] Salonen J., Laitinen L., Kaukonen A.M., Tuura J., Björkqvist M., Heikkilä T., Vähä-Heikkilä K., Hirvonen J., Lehto V.P. (2005). J. Control. Release..

[177] Salonen J., Kaukonen A.M., Hirvonen J., Lehto V.P. (2008). J. Pharm. Sci..

[178] Santos H.A., Salonen J., Bimbo L.M., Lehto V.P., Peltonen L., Hirvonen J. (2011). J. Drug Deliv. Sci. Tech..

[179] Wang F., Hui H., Barnes T.J., Barnett C., Prestidge C.A. (2010). Mol. Pharmaceutics..

[180] Zvyagin A.V., Song Z., Nadort A., Sreenivasan V.K.A., Deyev S.M. (2013). Handbook of Nano-Optics and Nanophotonics. Berlin, Heidelberg: Springer,.

[181] Tuchin V.V. (2016). J. Biomed. Photon. Eng..

[182] Min Y., Li J., Liu F., Padmanabhan P., Yeow E.K., Xing B. (2014). Nanomaterials (Basel)..

[183] Lingeshwar Reddy K., Balaji R., Kumar A., Krishnan V. (2018). Small..

[184] Song Z., Anissimov Y.G., Zhao J., Nechaev A.V., Nadort A., Jin D., Prow T.W., Roberts M.S., Zvyagin A.V. (2012). J. Biomed. Opt..

[185] Wen S., Zhou J., Zheng K., Bednarkiewicz A., Liu X., Jin D. (2018). Nat. Commun..

[186] Zhan Q., Qian J., Liang H., Somesfalean G., Wang D., He S., Zhang Z., Andersson-Engels S. (2011). ACS Nano..

[187] Singh R., Dumlupinar G., Andersson-Engels S., Melgar S. (2019). Int. J. Nanomed..

[188] Zhong Y., Tian G., Gu Z., Yang Y., Gu L., Zhao Y., Ma Y., Yao J. (2014). Adv. Mater..

[189] Zhou J., Liu Q., Feng W., Sun Y., Li F. (2015). Chem. Rev..

[190] Generalova A.N., Kochneva I.K., Khaydukov E.V., Semchishen V.A., Guller A.E., Nechaev A.V., Shekhter A.B., Zubov V.P., Zvyagin A.V., Deyev S.M. (2015). Nanoscale..

[191] Guryev E.L., Shilyagina N.Y., Kostyuk A.B., Sencha L.M., Balalaeva I.V., Vodeneev V.A., Kutova O.M., Lyubeshkin A.V., Yakubovskaya R.I., Pankratov A.A. (2019). Toxicol. Sci..

[192] Muhr V., Wilhelm S., Hirsch T., Wolfbeis O.S. (2014). Acc. Chem. Res..

[193] Oliveira H., Bednarkiewicz A., Falk A., Fröhlich E., Lisjak D., Prina-Mello A., Resch S., Schimpel C., Vrček I.V., Wysokińska E. (2019). Adv. Healthc. Mater..

[194] Chen F., Bu W., Cai W., Shi J. (2013). Curr. Mol. Med..

[195] Park Y.I., Lee K.T., Suh Y.D., Hyeon T. (2015). Chem. Soc. Rev..

[196] DaCosta M.V., Doughan S., Han Y., Krull U.J. (2014). Anal. Chim. Acta..

[197] Radunz S., Andresen E., Würth C., Koerdt A., Tschiche H.R., Resch-Genger U. (2019). Anal. Chem..

[198] Khaydukov E.V., Mironova K.E., Semchishen V.A., Generalova A.N., Nechaev A.V., Khochenkov D.A., Stepanova E.V., Lebedev O.I., Zvyagin A.V., Deyev S.M. (2016). Sci. Rep..

[199] Li P., Yan Y., Chen B., Zhang P., Wang S., Zhou J., Fan H., Wang Y., Huang X. (2018). Biomater. Sci..

[200] Grebenik E.A., Kostyuk A.B., Deyev S.M. (2016). Russ. Chem. Rev..

[201] Shanwar S., Liang L., Nechaev A.V., Bausheva D.K., Balalaeva I.V., Vodeneev V.A., Roy I., Zvyagin A.V., Guryev E.L. (2021). Materials..

[202] Shramova E.I., Kotlyar A.B., Lebedenko E.N., Deyev S.M., Proshkina G.M. (2020). Acta Naturae..

[203] Grebenik E.A., Nadort A., Generalova A.N., Nechaev A.V., Sreenivasan V.K.A., Khaydukov E.V., Semchishen V.A., Popov A.P., Sokolov V.I., Akhmanov A.S. (2013). J. Biomed. Optics..

[204] Grebenik E.A., Generalova A.N., Nechaev A.V., Khaydukov E.V., Mironova K.E., Stremovskiy O.A., Lebedenko E.N., Zvyagin A.V., Deyev S.M. (2014). Acta Naturae..

[205] Rocheva V.V., Savelyev A.G., Nechaev A.V., Generalova A.N., Semchishen V.A., Zvyagin A.V., Khaydukov E.V. (2019). Opt. Spectrosc..

[206] Polikarpov D., Liang L., Care A., Sunna A., Campbell D., Walsh B., Balalaeva I.V., Zvyagin A.V., Gillatt D., Guryev E.L. (2019). Biomolecules..

[207] Guryev E.L., Smyshlyaeva A.S., Shilyagina N.Y., Shanwar S., Kostyuk A.B., Shulga A.A., Konovalova E.V., Zvyagin A.V., Deyev S.M., Petrov R.V. (2020). Dokl. Biochem. Biophysic..

[208] Guryev E.L., Smyshlyaeva A.S., Shilyagina N.Y., Sokolova E.A., Shanwar S., Kostyuk A.B., Lyubeshkin A.V., Schulga A.A., Konovalova E.V., Lin Q., Roy I., Balalaeva I.V., Deyev S.M., Zvyagin A.V. (2020). Molecules..

[209] Guryev E.L., Volodina N.O., Shilyagina N.Yu., Gudkov S.V., Balalaeva I.V., Volovetskii A.B., Lyubeshkin A.V., Sen A.V., Ermilov S.A., Vodeneev V.A. (2018). Proc. Natl. Acad. Sci. USA..

[210] Liang L., Care A., Zhang R., Lu Y., Packer N.H., Sunna A., Qian Y., Zvyagin A.V. (2016). ACS Appl. Mat. Interfaces..

[211] Mironova K.E., Khochenkov D.A., Generalova A.N., Rocheva V.V., Sholina N.V., Nechaev A.V., Semchishen V.A., Deyev S.M., Zvyagin A.V., Khaydukov E.V. (2017). Nanoscale..

[212] Nikitin M.P., Zelepukin I.V., Shipunova V.O., Sokolov I.L., Deyev S.M., Nikitin P.I. (2020). Nat. Biomed. Eng..

[213] Zelepukin I.V., Yaremenko A.V., Yuryev M.V., Mirkasymov A.B., Sokolov I.L., Deyev S.M., Nikitin P.I., Nikitin M.P. (2020). J. Cont. Release..

[214] Shilova O.N., Deyev S.M. (2019). Acta Naturae..

